# Supplementary material for: Modeling glioblastoma heterogeneity as a dynamic network of cell states
Source: Mol Syst Biol. 2021 Sep 16;17(9):e10105. doi: 10.15252/msb.202010105 (PMC8444284; doi:10.15252/msb.202010105)
Supplement: Supplementary file 5 — Source Data for Figure 3 [file MSB-17-e10105-s001.zip › Figure3A_sourcedata/GSEA_3065/nosigdb_state5.GseaPreranked.1622718435914/NB_KI_CLUSTER5.html]

Details for gene set NB\_KI\_CLUSTER5[GSEA]

|  || Dataset | state5 |
| Phenotype | NoPhenotypeAvailable |
| Upregulated in class | na\_neg |
| GeneSet | NB\_KI\_CLUSTER5 |
| Enrichment Score (ES) | -0.5316595 |
| Normalized Enrichment Score (NES) | -1.8133575 |
| Nominal p-value | 0.0 |
| FDR q-value | 0.0018045893 |
| FWER p-Value | 0.044 |
Table: GSEA Results Summary

  

Fig 1: Enrichment plot: NB\_KI\_CLUSTER5      
 Profile of the Running ES Score & Positions of GeneSet Members on the Rank Ordered List

  

| PROBE | GENE SYMBOL | GENE\_TITLE | RANK IN GENE LIST | RANK METRIC SCORE | RUNNING ES | CORE ENRICHMENT || 1 | GGH |  |  | 184 | 0.453 | 0.0019 | No |
| 2 | LBR |  |  | 198 | 0.440 | 0.0223 | No |
| 3 | ARL6IP1 |  |  | 232 | 0.418 | 0.0394 | No |
| 4 | HELLS |  |  | 280 | 0.393 | 0.0537 | No |
| 5 | CDCA7 |  |  | 495 | 0.313 | 0.0452 | No |
| 6 | BRCA1 |  |  | 522 | 0.305 | 0.0574 | No |
| 7 | FANCA |  |  | 559 | 0.295 | 0.0681 | No |
| 8 | DDX11 |  |  | 1117 | 0.196 | 0.0153 | No |
| 9 | NASP |  |  | 1225 | 0.182 | 0.0123 | No |
| 10 | CEP152 |  |  | 1436 | 0.159 | -0.0034 | No |
| 11 | MIS18BP1 |  |  | 1765 | 0.129 | -0.0338 | No |
| 12 | KIF20B |  |  | 1962 | 0.114 | -0.0502 | No |
| 13 | MXD3 |  |  | 2178 | 0.100 | -0.0694 | No |
| 14 | DIAPH3 |  |  | 2387 | 0.086 | -0.0884 | No |
| 15 | ATAD2 |  |  | 2545 | 0.077 | -0.1023 | No |
| 16 | CHEK1 |  |  | 2877 | 0.059 | -0.1366 | No |
| 17 | LIG1 |  |  | 3181 | 0.042 | -0.1685 | No |
| 18 | SMC2 |  |  | 3374 | 0.032 | -0.1885 | No |
| 19 | CIP2A |  |  | 3713 | 0.020 | -0.2255 | No |
| 20 | NCAPD2 |  |  | 3914 | 0.011 | -0.2474 | No |
| 21 | PRC1 |  |  | 4241 | -0.003 | -0.2839 | No |
| 22 | TCF19 |  |  | 4635 | -0.020 | -0.3271 | No |
| 23 | CENPK |  |  | 4675 | -0.021 | -0.3304 | No |
| 24 | SGO2 |  |  | 4709 | -0.022 | -0.3330 | No |
| 25 | CDK1 |  |  | 4766 | -0.025 | -0.3381 | No |
| 26 | PRR11 |  |  | 4822 | -0.026 | -0.3429 | No |
| 27 | SHCBP1 |  |  | 4848 | -0.027 | -0.3444 | No |
| 28 | EZH2 |  |  | 4858 | -0.028 | -0.3440 | No |
| 29 | MCM7 |  |  | 4986 | -0.032 | -0.3567 | No |
| 30 | GINS2 |  |  | 5024 | -0.034 | -0.3592 | No |
| 31 | CDT1 |  |  | 5191 | -0.040 | -0.3758 | No |
| 32 | ECT2 |  |  | 5264 | -0.043 | -0.3818 | No |
| 33 | SPDL1 |  |  | 5460 | -0.051 | -0.4012 | No |
| 34 | HIST2H2AC |  |  | 5542 | -0.055 | -0.4076 | No |
| 35 | TRIP13 |  |  | 5570 | -0.056 | -0.4078 | No |
| 36 | BUB3 |  |  | 5596 | -0.057 | -0.4078 | No |
| 37 | CLSPN |  |  | 5614 | -0.058 | -0.4068 | No |
| 38 | KPNA2 |  |  | 5811 | -0.066 | -0.4255 | No |
| 39 | RACGAP1 |  |  | 5902 | -0.070 | -0.4322 | No |
| 40 | CHAF1A |  |  | 6157 | -0.082 | -0.4566 | No |
| 41 | CENPM |  |  | 6368 | -0.093 | -0.4756 | No |
| 42 | CKAP2 |  |  | 6372 | -0.094 | -0.4712 | No |
| 43 | ZWINT |  |  | 6453 | -0.099 | -0.4753 | No |
| 44 | DNMT1 |  |  | 6455 | -0.099 | -0.4705 | No |
| 45 | SMC4 |  |  | 6517 | -0.102 | -0.4723 | No |
| 46 | MCM5 |  |  | 6553 | -0.105 | -0.4710 | No |
| 47 | DBF4 |  |  | 6572 | -0.106 | -0.4677 | No |
| 48 | PLK1 |  |  | 6581 | -0.107 | -0.4633 | No |
| 49 | ANP32E |  |  | 6655 | -0.111 | -0.4659 | No |
| 50 | FBXO5 |  |  | 6775 | -0.119 | -0.4734 | No |
| 51 | ZWILCH |  |  | 6812 | -0.122 | -0.4714 | No |
| 52 | CDC25B |  |  | 7102 | -0.146 | -0.4966 | No |
| 53 | CENPW |  |  | 7116 | -0.147 | -0.4908 | No |
| 54 | GMNN |  |  | 7224 | -0.158 | -0.4949 | No |
| 55 | MCM3 |  |  | 7552 | -0.198 | -0.5218 | Yes |
| 56 | UBE2T |  |  | 7561 | -0.199 | -0.5128 | Yes |
| 57 | BIRC5 |  |  | 7596 | -0.204 | -0.5065 | Yes |
| 58 | TEX30 |  |  | 7639 | -0.209 | -0.5008 | Yes |
| 59 | CDKN2D |  |  | 7721 | -0.222 | -0.4989 | Yes |
| 60 | PCLAF |  |  | 7734 | -0.223 | -0.4892 | Yes |
| 61 | MZT1 |  |  | 7838 | -0.242 | -0.4887 | Yes |
| 62 | DHFR |  |  | 7878 | -0.250 | -0.4806 | Yes |
| 63 | CKS2 |  |  | 7972 | -0.270 | -0.4776 | Yes |
| 64 | HMGB2 |  |  | 7973 | -0.271 | -0.4642 | Yes |
| 65 | HIST1H1C |  |  | 7978 | -0.271 | -0.4511 | Yes |
| 66 | PCNA |  |  | 8082 | -0.293 | -0.4481 | Yes |
| 67 | DUT |  |  | 8118 | -0.300 | -0.4371 | Yes |
| 68 | HIST1H1E |  |  | 8176 | -0.320 | -0.4276 | Yes |
| 69 | CKS1B |  |  | 8241 | -0.336 | -0.4180 | Yes |
| 70 | TUBB4B |  |  | 8356 | -0.371 | -0.4124 | Yes |
| 71 | NUDT1 |  |  | 8359 | -0.373 | -0.3940 | Yes |
| 72 | PTTG1 |  |  | 8446 | -0.406 | -0.3835 | Yes |
| 73 | NUCKS1 |  |  | 8502 | -0.435 | -0.3680 | Yes |
| 74 | RAN |  |  | 8555 | -0.463 | -0.3508 | Yes |
| 75 | HMGN2 |  |  | 8608 | -0.496 | -0.3320 | Yes |
| 76 | HMGB1 |  |  | 8653 | -0.527 | -0.3107 | Yes |
| 77 | ANP32B |  |  | 8680 | -0.545 | -0.2865 | Yes |
| 78 | H2AFV |  |  | 8693 | -0.554 | -0.2602 | Yes |
| 79 | RHEB |  |  | 8694 | -0.554 | -0.2327 | Yes |
| 80 | DEK |  |  | 8770 | -0.627 | -0.2099 | Yes |
| 81 | H2AFZ |  |  | 8774 | -0.630 | -0.1788 | Yes |
| 82 | RANBP1 |  |  | 8819 | -0.669 | -0.1505 | Yes |
| 83 | HIST1H4C |  |  | 8850 | -0.710 | -0.1186 | Yes |
| 84 | UBE2S |  |  | 8901 | -0.772 | -0.0858 | Yes |
| 85 | GAPDH |  |  | 8915 | -0.803 | -0.0473 | Yes |
| 86 | TUBA1B |  |  | 8980 | -1.103 | 0.0004 | Yes |
Table: GSEA details [plain text format]

  

Fig 2: NB\_KI\_CLUSTER5: Random ES distribution      
 Gene set null distribution of ES for **NB\_KI\_CLUSTER5**
